# Shear Strength Prediction Model for RC Exterior Joints Using Gene Expression Programming

**DOI:** 10.3390/ma15207076

**Published:** 2022-10-12

**Authors:** Moiz Tariq, Azam Khan, Asad Ullah

**Affiliations:** NUST Institute of Civil Engineering (NICE), Sector H-12, Islamabad 44000, Pakistan

**Keywords:** gene expression programming (GEP), reinforce concrete, exterior joint, shear strength

## Abstract

Predictive models were developed to effectively estimate the RC exterior joint’s shear strength using gene expression programming (GEP). Two separate models are proposed for the exterior joints: the first with shear reinforcement and the second without shear reinforcement. Experimental results of the relevant input parameters using 253 tests were extracted from the literature to carry out a knowledge analysis of GEP. The database was further divided into two portions: 152 exterior joint experiments with joint transverse reinforcements and 101 unreinforced joint specimens. Moreover, the effects of different material and geometric factors (usually ignored in the available models) were incorporated into the proposed models. These factors are beam and column geometries, concrete and steel material properties, longitudinal and shear reinforcements, and column axial loads. Statistical analysis and comparisons with previously proposed analytical and empirical models indicate a high degree of accuracy of the proposed models, rendering them ideal for practical application.

## 1. Introduction

The failures of reinforced concrete structures during earthquakes seem to mostly occur at the connections of main structural elements. This is due to the complexity of the forces generated during the cyclic loadings at the joints of the supporting members [1,2,3,4,5,6,7,8,9,10]. In this respect, the shear failure of the beam–column joints was noted as the principal cause of the collapse of RC frame buildings in recent earthquakes. Furthermore, limited guidance provided by well-established design codes justifies the need for further investigating the shear study of RC joints [11,12].

Post-earthquake damage assessments have attributed the shear failure of the beam–column joints to inadequate joint confinement. Several joint failures have identified the inadequacy of building codes during recent earthquakes (Izmit 1999 [13], Tehuacan 1999 [14], Chi-Chi 1999 [15], and Kashmir 2005 [16]). The seismic damages observed after the L’Aquila earthquake are of special interest, which indicated severe structural deficiencies in the RC joint, either due to the lack of a capacity design approach or poor reinforcement detailing.

Building codes governing the design practices in most countries (New Zealand code [17], Eurocode [18], Chinese code [19], Japanese code [20], ACI318-19 [21], ACI352R-02 [22], and ASCE 41-17 [23]) propose single design formulas for determining the shear strengths of the exterior joints, associated with the concrete compressive strength only. However, different experimental tests and analytical models indicate that compressive strength alone is not the governing factor. Owing to the discontinuation of any beam at the exterior joint and the ensuing complexity, the shear strength expression demands the incorporation of governing factors, such as the concrete compressive strength, joint geometry, joint shear reinforcement, the beam longitudinal reinforcement ratio, and the axial column load. Therefore, the industry is seeking a cost-effective solution that incorporates the key influencing parameters and predicts the exterior joint shear strength accurately.

Many analytical and computational models for both the interior and the exterior joints have been developed. For example, the modified compression field theory has been proposed by Hwang et al. [24]. There was also a strut and tie model proposed by Hsu et al. [25] that has been modified by Wong et al. [26] and Pang and Hsu [27]. Similarly, Pauletta et al. [28] have proposed an exterior joint shear strength model using the strut-and-tie analogy. Nevertheless, all of these models demand personal judgments based on collective analyses and design experience.

To approximately describe the behaviors of exterior joints, Vollum et al. [29] proposed an equation based on the joint aspect ratio and the concrete compressive strength. However, this equation applies to the structural assemblies subjected to monotonic loading only. Further investigation on the joint shear strength has been carried out by Bakir et al. [4], who proposed an expression including the longitudinal reinforcement and the concrete compressive strength. Keeping all the factors the same, as prescribed by Bakir et al. [4], and replacing the beam longitudinal reinforcement with the longitudinal column reinforcement, another model was developed by Hegger et al. [30]. Similar simplified empirical models, subsuming various influencing parameters, were proposed by different authors (Sarsam et al. [31], Parra-Montesinos et al. [32] and Kim et al. [33]. It is important to note that most of the empirical models proposed in the literature are based on the reinforced exterior joint, which can significantly overestimate the joint capacity if applied to unreinforced joints. In addition, a limited number of models have been cross-validated with experiments. Therefore, in view of the available experimental data, a reassessment of the design theory of the reinforced and the unreinforced exterior joints is important for the precise assessment of the shear failure.

This study aims to develop a precise and robust model for the shear strength of the RC beam–column exterior joint by employing the gene expression programming (GEP) tool. An extensive database of 254 experiments was used to analyze and validate the proposed GEP model. This model was applied to both the reinforced and the unreinforced exterior joints. Finally, the outcomes of the proposed equation are statistically compared to other models derived from the literature.

## 2. Research Significance

RC joints are a subject of interest for many researchers because their failure threatens the structural integrity of structures, especially during seismic events. According to ACI 352R-02, joints are considered the extended parts of the columns connected with the beams in a frame system. The inadequate shear capacity of columns increases seismic vulnerability, leading to brittle shear failure [29,30,31,32,33,34,35,36,37,38,39,40,41,42,43,44]. This notion of the RC joint as an extension of the column also makes the columns vulnerable to shear failure, especially under seismic loading. From this precedent, considering joints as rigidly fixed connections in structural design practices is not advisable. In the context of seismic loading, the RC beam–column joint follows a transfer of complex forces within the joint. Thus, the mobilization of stress between the main structural elements needs the proper design of RC joints based on their nominal strength.

Consequently, various structural design codes and researchers have focused on predicting the nominal shear capacity of exterior RC joints. Nevertheless, it may be inferred from the literature survey that these approaches do not capture the essence of the complex shear response of RC joints. This is because most of the available models tend to ignore the key influencing parameters, such as beam and column geometries, concrete and steel material properties, longitudinal and shear reinforcements, and column axial loads. The present study aimed to develop a GEP-based shear prediction model incorporating all key factors in light of the preceding discussion.

## 3. Basic Mechanics

**Figure 1:**
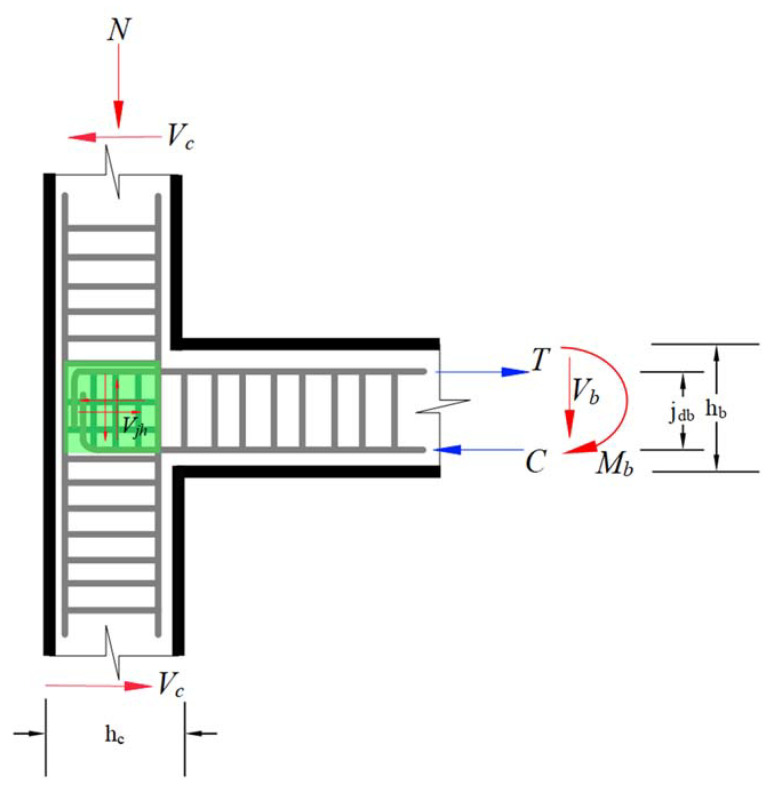
Cyclically-loaded beam–column joint.

RC concrete joints experience a significant amount of shear force in the joint cores [45]. Figure 1 represents the mechanics of the external beam–column joint subjected to the cyclic load.

Plastic hinges are expected to develop in the beams adjacent to the column face when subjected to seismic action. Similarly, the shear in the joint panel is related to developing the high shear stress in the column and the portion of the tensile force in the longitudinal reinforcing bars. So, the maximum horizontal shear force, *V_jh_*, in the joint panel is computed as follows:

(1) $$V_{jh}=T-V_{c}=A_{s}f_{y}-V_{c}=\frac{M_{b}}{jd_{b}}-V_{c}$$

where $V_{jh}$ and $V_{c}$ represent the shear stress in the column and joint panel, respectively, T shows the tension in the beam’s longitudinal reinforcement. $M_{b}$ shows the moment in the beam member due to the cyclic load and $\;jd_{b}$ represents the moment arm between the beam tensile and compression zone. The nominal shear strength of the joint region is represented by Equation (2),

(2) $$V_{n}=V_{jh}=V_{ch}+V_{sh}$$

where $V_{ch}$ and $V_{sh}$ represent the shear component of concrete and the joint transverse reinforcement, respectively.

The exterior joint shear strength was estimated using a variety of formulas in the literature. These equations are based on experiments, but it is surprising to see such a wide range of results. Moreover, there is a wide range of shear strength prediction equations in design codes of practice. Various joint models are briefly reviewed in Appendix A.

## 4. Fundamentals of Gene Expression Programming

Gene expression programming (GEP) is a popular evolutionary algorithm, which can process the input data in a domain-independent way. The GEP can represent chromosomes in the form of linear and non-linear strings of different sizes and shapes. This quality of GEP contributes to better performance compared to other algorithms, such as genetic programming (GP) and a genetic algorithm (GA).

The GEP algorithm conducts several trials by altering the performance parameters that include the number of genes, head size, chromosomes, and linking functions. In this way, the GEP generates an optimized solution based on the supplied input population. However, a rare predicament encountered by the authors during the GEP algorithm operation is the inability to reach the best global solution, thereby either leading to undefined steps or giving an illogical expression. This problem can be solved by changing the number of genes and chromosomes, or by changing the way they link together.

Because of the aforementioned advantages, the GEP has become very popular in the structural engineering industry over the last decade. GEP has shown promise in developing empirical equations for various structural components. For example, various RC structural element capacities have been fruitfully predicted by the GEP expressions [46,47,48].

### Experimental Database

An extensive database of 256 experiments [1,7,25,49,50,51,52,53,54,55,56,57,58,59,60,61,62,63,64,65,66,67,68,69,70,71,72,73,74,75,76,77,78,79,80,81,82,83,84,85,86,87,88,89,90,91,92,93,94,95,96,97,98,99,100,101,102,103] has been compiled for estimating the shear strength of reinforced and unreinforced RC exterior joints under cyclic loading. Out of this dataset, 156 experiments have shear reinforcements in the exterior joints, while 100 experiments have no shear reinforcements. A random portion of the 256 experiments was selected as calibration data to improve the models; the remaining were used as validation data. Table 1 summarizes the ranges of the database used to extract the model for both the reinforced and unreinforced exterior joints. The major categories within this database included the concrete compressive strength, the joint transverse reinforcement, the column depth, the joint panel width, the beam reinforcement ratio, and the column axial load, as shown in Table 1. Early studies have shown that increasing the concrete compressive strength and the joint shear reinforcement improves the joint shear strength. In contrast, increasing the joint aspect ratio decreases the joint shear strength [78,79]. Of special interest is that the configuration of the transverse joint reinforcement has a particularly large effect on the joint shear strength [104]. Moreover, the rectangular spiral reinforcement provides better shear capacity than the typical stirrups [104].

## 5. Proposed GEP Model for Estimating Joint Shear

**Figure 2:**
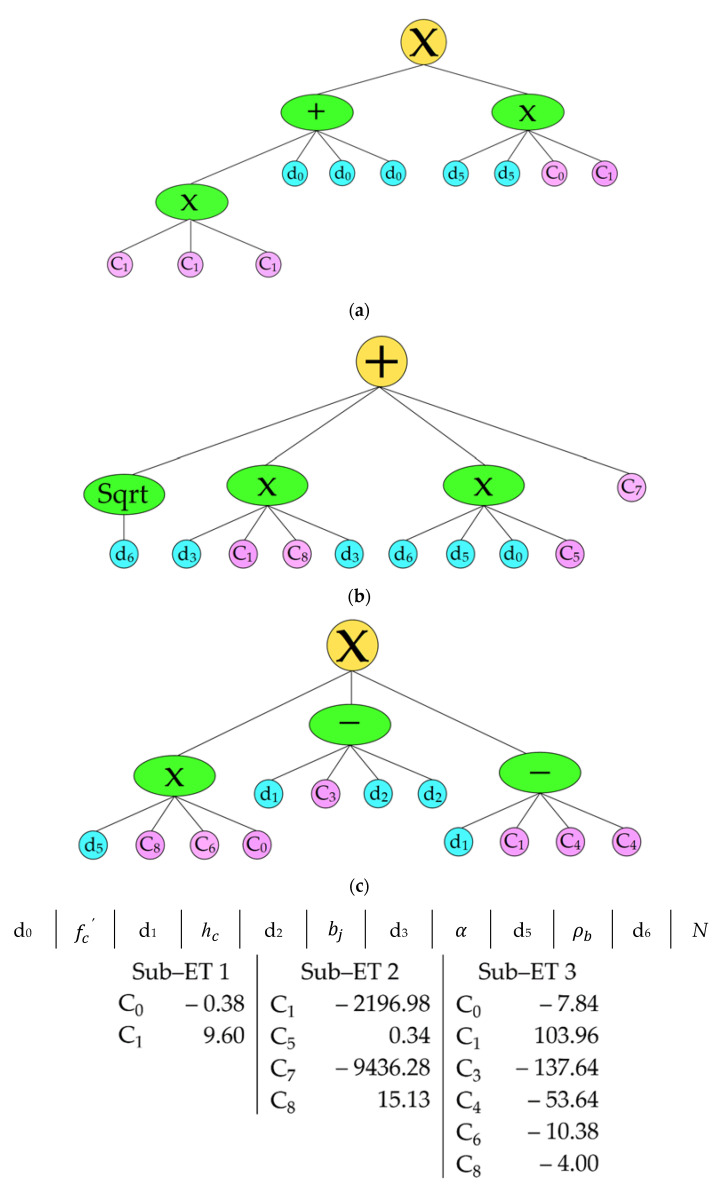
Gene expression tree for the unreinforced exterior joint. (**a**) Sub–ET 1 (**b**) Sub–ET 2 (**c**) Sub–ET 3.

**Figure 3:**
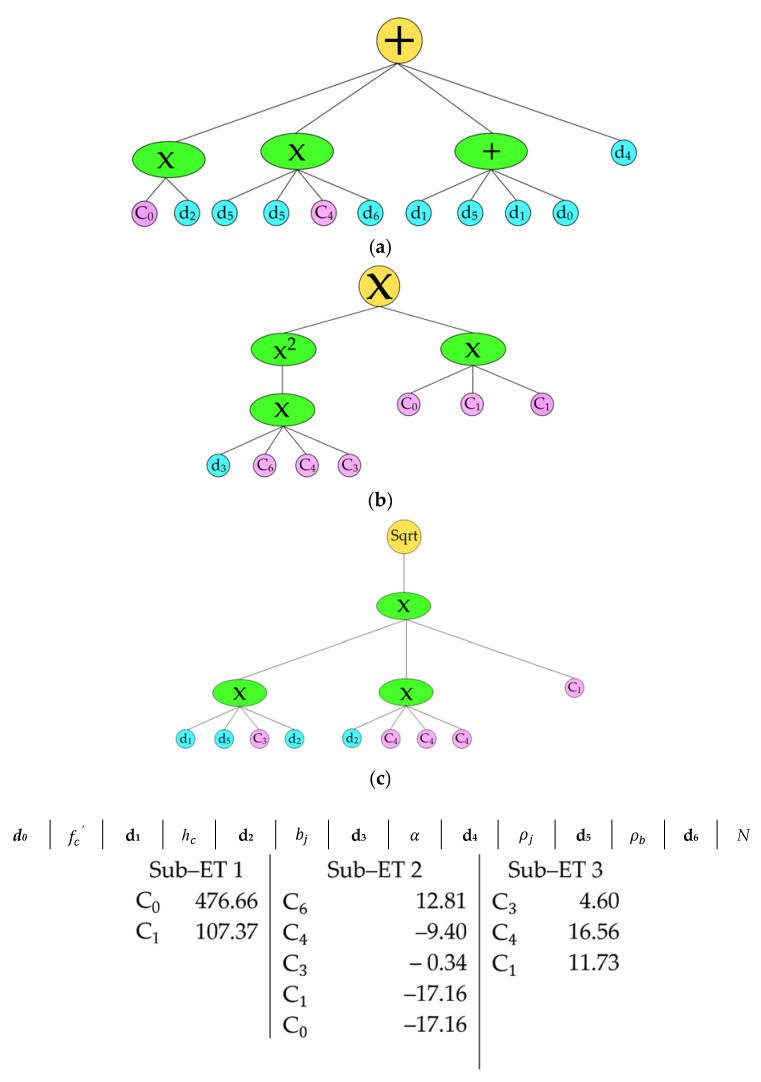
Gene expression tree for the reinforced exterior joint. (**a**) Sub–ET 1 (**b**) Sub–ET 2 (**c**) Sub–ET 3.

In this section, we aimed to construct a gene expression programming (GEP) model for both the reinforced and the unreinforced exterior joints using the Gene Xpro tool. The gene expression tree is given in Figure 2 and Figure 3. A simplified model was produced through straightforward linking functions, e.g., addition, subtraction, multiplication, and division. In addition, a step-by-step increase in the number of genes and head size, whilst keeping the increase to the minimum, also ensured the generation of a simpler model. The model construction parameter is presented in Table 2. The following simple relationships can represent the GEP models generated from the previously mentioned dataset:

For the unreinforced exterior joint,

(3) $$V_{{\mathrm{jh}}_{\mathrm{NS}}}{=V}_{1\mathrm{NS}}{+V}_{2\mathrm{NS}}{+V}_{3\mathrm{NS}}$$

(4) $$V_{1\mathrm{NS}\;}=\left(92{.16h}_{c}{+3f}_{c}{}^{\prime }\right)\;\times \;\left({2\rho }_{b}+9.221\right)$$

(5) $$V_{2\mathrm{NS}}=\left(\sqrt{N}\;{-\;32940\alpha }^{2}+0{.34N\rho }_{b}f_{c}{}^{\prime }\right)\;-\;9436.28$$

(6) $$V_{3\mathrm{NS}}=68364{.49h}_{c}\rho_{b}\left(h_{c}\;{-\;2b}_{j}+137.64\right)$$

For the reinforced exterior joint,

(7) $$V_{{\mathrm{jh}}_{S}}{=V}_{1S}{+V}_{2S}{+V}_{3S}$$

(8) $$V_{1S}=\left(476{.65b}_{j\;}+107{.37\;\rho }_{b}{}^{2}{N+2h}_{c}\rho_{b}f_{c}{+\rho }_{j}\right)$$

(9) $$V_{2S}=5816.10{\left(\alpha +3.025\right)}^{2}$$

(10) $$V_{3S}{=246894h}_{c}\rho_{b}b_{j}$$

where $h_{c}$ defines the depth of the column member, $b_{j}$ represents the effective joint width, $\rho_{b}$ represents the longitudinal tensile reinforcement, respectively, $f_{c}\prime$ defines the concrete compressive strength, $\rho_{j}$ represents the joint shear reinforcement area, α shows the joint aspect ratio, and N represents the axial load on the column.

**Figure 4:**
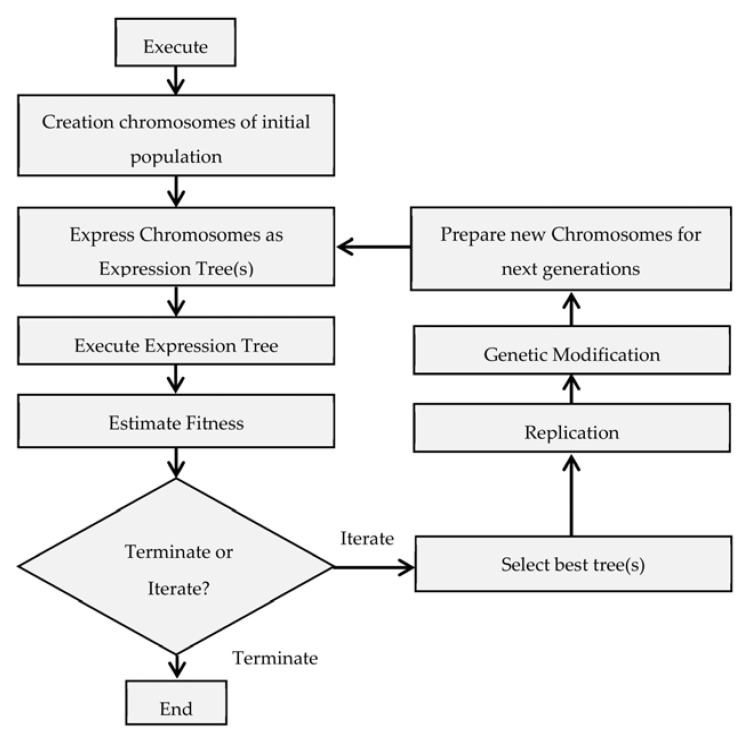
Flowchart demonstrating the model selection in gene expression programming.

The GEP optimization is shown in Figure 4. The GEP process involves many important steps, including the function set, the terminal set, the fitness function, the control parameters, and the stop condition. Initially, a fitness function was set up. Then a random string of chromosomes was generated into an expression tree that looked similar to a mathematical expression. The fitness score of these chromosomes was evaluated so that the best solutions could be found [104].

## 6. Statistical Validation of the Developed Model

Validation of the GEP model is important for assessing its predictive capability. For this purpose, experimental and statistical validations were performed to ensure the validity of the proposed model. For experimental validation, the test performed by Bindhu et al. [77] was selected as a benchmark test, which was not included in either the model generation or validation. An illustrative example is presented in Appendix B. However, for statistical validation, 60% of the randomly selected samples of reinforced and unreinforced RC joints were used for the model calibration, and the rest of the 40% was used for the model validation. Subsequently, statistical validation was carried out to make sure that the predictive model worked correctly. Some of these performance parameters are discussed below.

The coefficient of variation (*CoV)* checks the data spread using the following expression:

(11) $$\mathrm{CoV}\;\left(\%\right)=\left\{\frac{\mathrm{Standard}\;\mathrm{Devation}\;\left(\sigma \right)}{\mathrm{Mean}\;\left(\mu \right)}\right\}\times 100$$

The average absolute error (*AAE*) in n number of test specimens was checked using the following expression:

(12) $$\mathrm{AAE}\;\left(\%\right)=\frac{1}{n}\sum \left[\frac{\left|V_{jh}^{Exp}-V_{jh}^{Est}\right|}{V_{jh}^{Exp}}\right]$$

The coefficient of determination ($R^{2}$) measures the data spread using:

(13) $$R^{2}=1-\frac{\sum {\left[V_{jh}^{Exp}-V_{jh}^{Est}\right]}^{2}}{\sum {\left[V_{jh}^{Exp}-V_{jh_{\left(Mean\right)}}^{Exp}\right]}^{2}}$$

**Figure 5:**
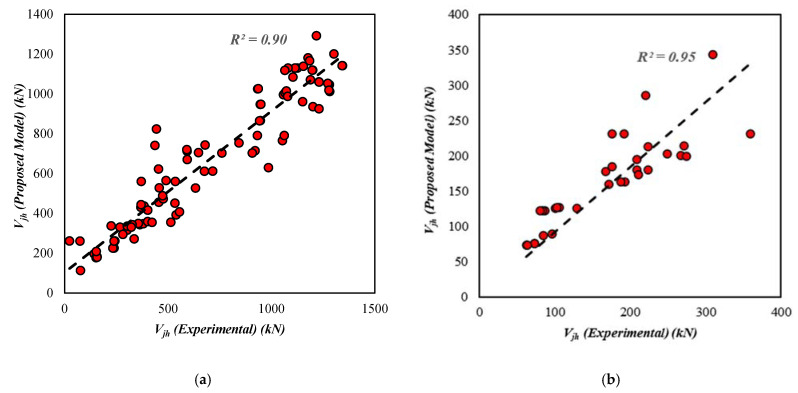

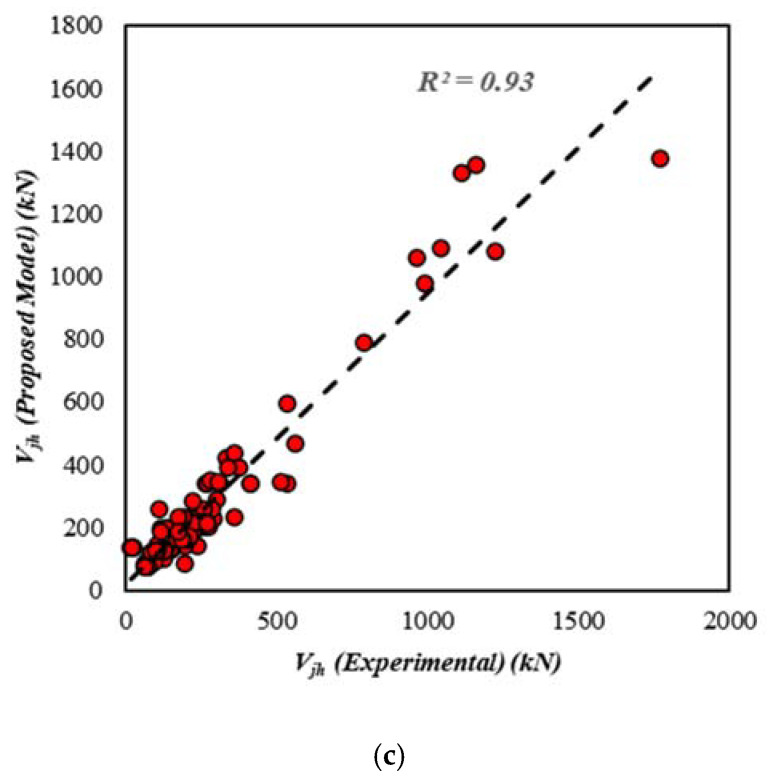
Results for the shear strength of the exterior joint (**a**) training data, (**b**) validation data, (**c**) all data.

**Figure 6:**
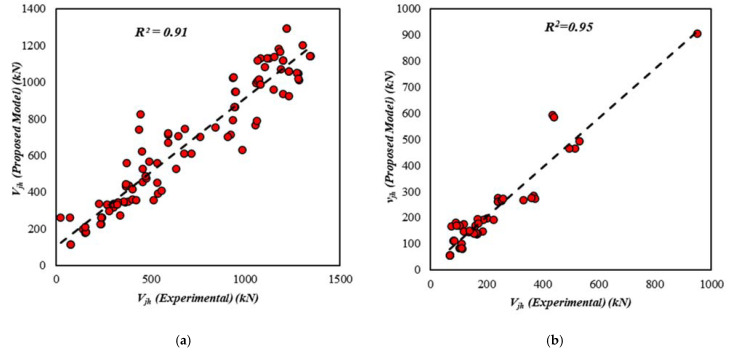

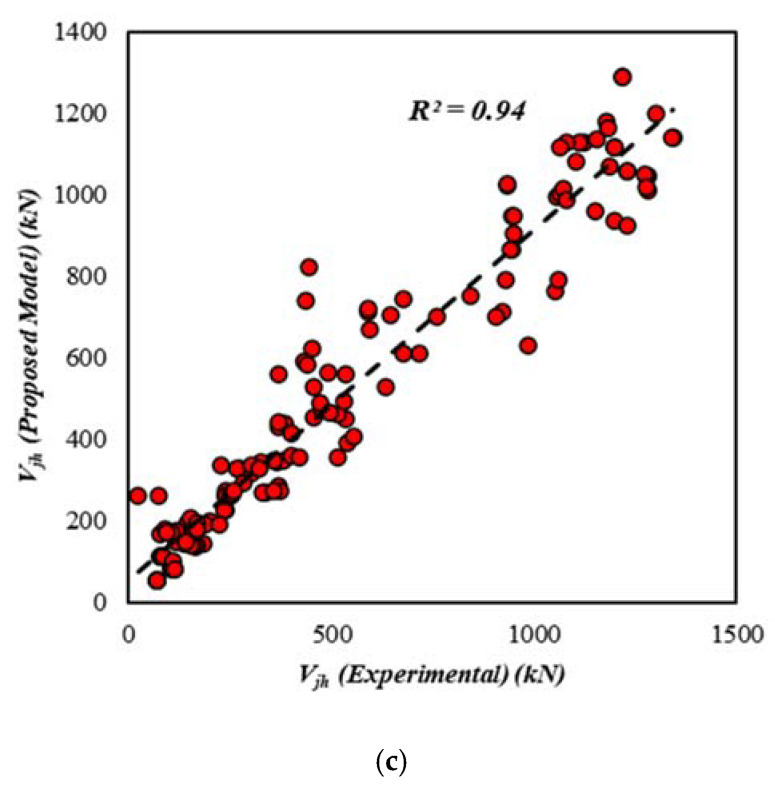
Results for the shear strength of the reinforced interior joints (**a**) training data, (**b**) validation data, (**c**) all data.

Figure 5 and Figure 6 show the coefficients of determination $R^{2}$ for both the unreinforced and reinforced exterior joint shear strength GEP models, respectively. The unreinforced joint model has a coefficient of determination of 0.90 for the training set, 0.95 for the validation set, and 0.93 for all data, as shown in Figure 5. Figure 6 shows that the coefficients of determination $R^{2}$ for the reinforced exterior joint model are 0.91 for training, 0.95 for validation data, and 0.94 for overall data. These values clearly show a strong correlation since $R^{2}$ is close to unity.

**Figure 7:**
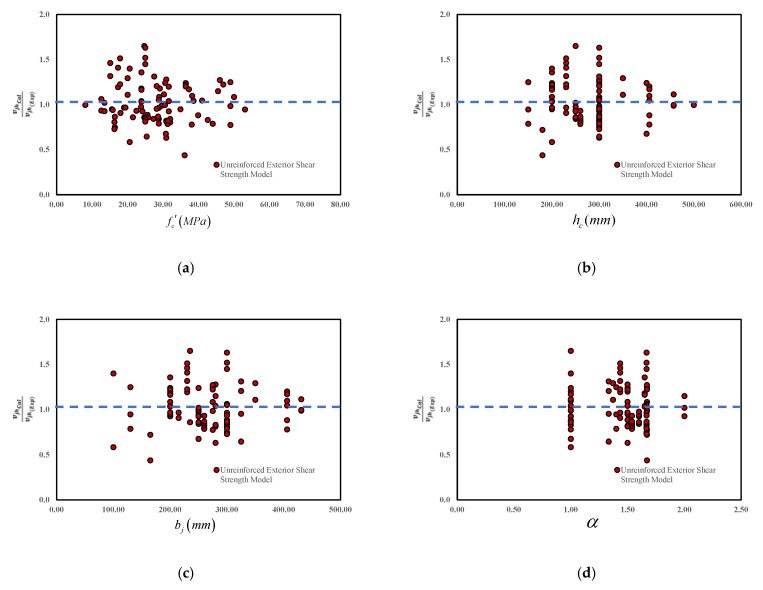

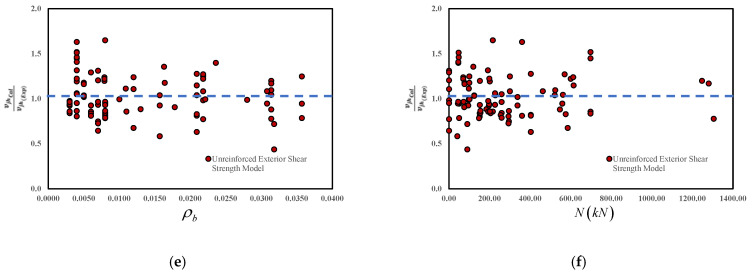
The predictive performance of the proposed unreinforced joint shear strength model based on the main parameters. (**a**) Concrete compressive Strength (**b**) Column Depth (**c**) Joint Width (**d**) Joint aspect ratio (**e**) Beam longitudinal reinforcement ratio (**f**) Joint Axial Load.

**Figure 8:**
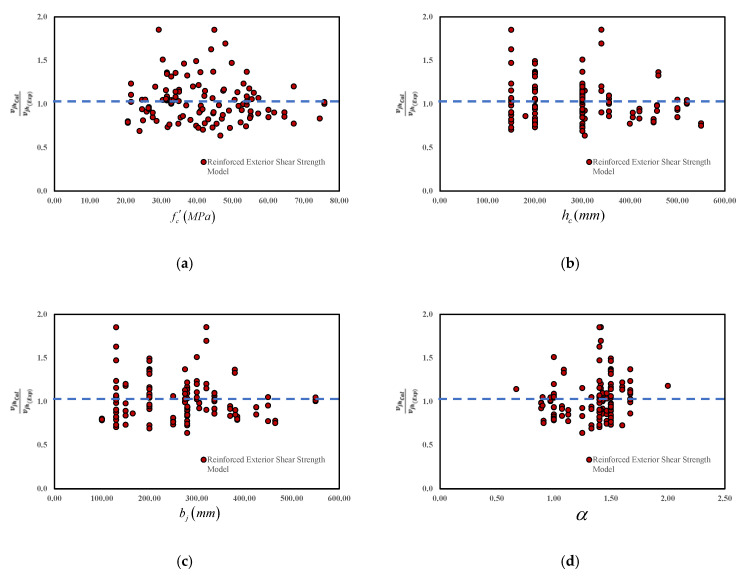

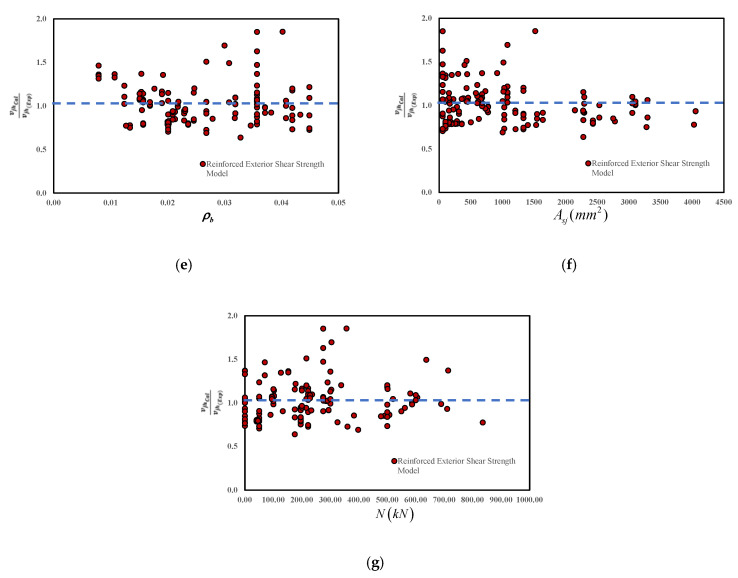
The predictive performance of the proposed reinforced joint shear strength model based on the main parameters. (**a**) Concrete compressive Strength (**b**) Column Depth (**c**) Joint Width (**d**) Joint aspect ratio (**e**) Beam longitudinal reinforcement ratio (**f**) Joint reinforcement area (**g**) Joint Axial Load.

The predictive ability of the proposed model is tested with different levels of the main influencing factors. For both cases of unreinforced and reinforced joints, the variation of the shear strength with $f_{c}{}^{\prime }$, $h_{b}$, $b_{j}$, $A_{sj}$, $\rho_{b}$, and *N* is demonstrated in Figure 7 and Figure 8. Figure 7a and Figure 8a indicate the accuracy of the model as a function of the concrete compressive strength with the virtual average of 1.00. Moreover, Figure 7 and Figure 8 represent the suitable model performances at various levels of $f_{c}{}^{\prime }$. Similarly, Figure 7b–d and Figure 8b–d show similar predictive performances based on the geometric properties, such as $h_{b}$ $b_{j}$, and α (aspect ratio). Even though a slight overestimation is observed, the overall performance is reasonably accurate within the interval [0.3–1.8]. A similar trend is also observed in Figure 7e and Figure 8e for the longitudinal reinforcement ($\rho_{b}$), Figure 7f and Figure 8f for the axial column load (*N*), and in Figure 8g for the joint shear reinforcement ($A_{sj}$). Consequently, it indicates that the proposed model has a relatively better performance than other available models.

## 7. Results and Discussions

A comparison is made herein between the developed GEP model and various existing empirical models. An additional comparison is made between the model and expressions proposed by several design codes of practice. Table 3 and Table 4 show the statistical comparisons of different models based on a complete set of 256 experiments.

### 7.1. Shear Strength of the Unreinforced Exterior Joint

**Figure 9:**
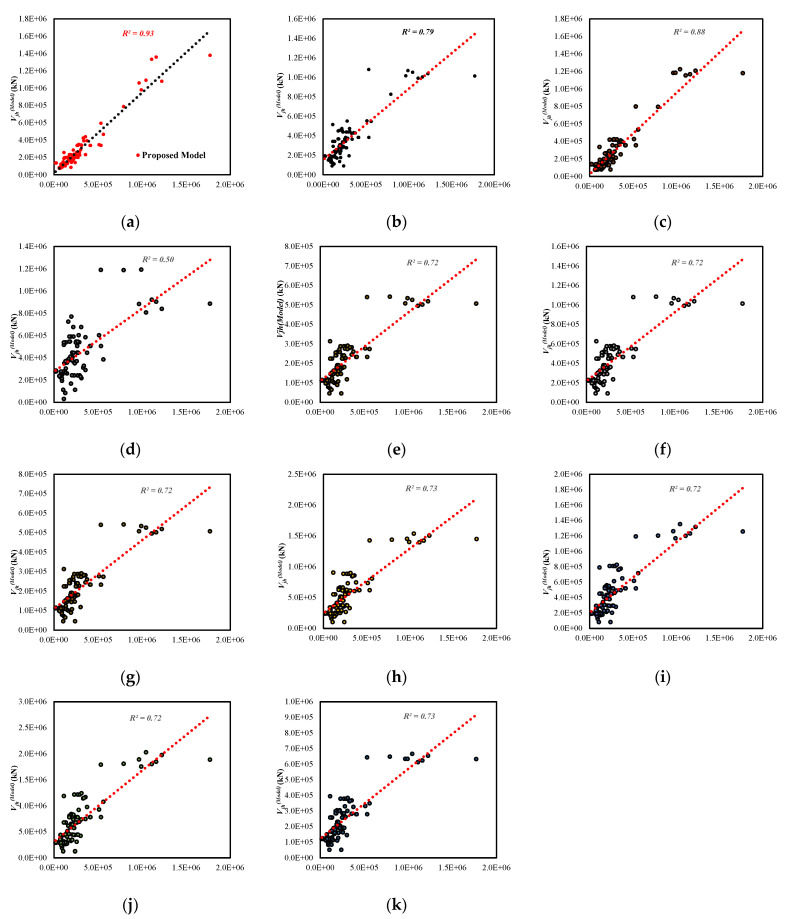
Comparison of the unreinforced RC exterior joint: (**a**) Proposed Equation, (**b**) Vollum et al. [24] (**c**) Bakir et al. [4] (**d**) Montenious et al. [27] (**e**) Kim et al. [28], (**f**) ACI 352R-02 [22], (**g**) ASCE 41-17 [23] (**h**) Eurocode [18] (**i**) New Zealand code [17] (**j**) British code [19] (**k**) Japanese code [20].

The GEP model under consideration has a higher coefficient of determination ($R^{2}$) than any of the other models in Table 1. The proposed model has a coefficient of determination ($R^{2}$) that is higher than the model developed by Vollum et al. [29]. Similarly, the proposed model performs better than the single factor-dependent models [4,17,18,19,20,21,22,23,24,25,26,27]. The proposed model’s average absolute error (*AAE*) is shown at 38%, which manifests the accuracy of the model. Likewise, the proposed equation yields the performance factor (PF) of 1.2, confirming the model’s efficiency. Figure 9 further substantiates the model’s reliability and validity.

### 7.2. Shear Strength of the Reinforced Exterior Joint

**Figure 10:**
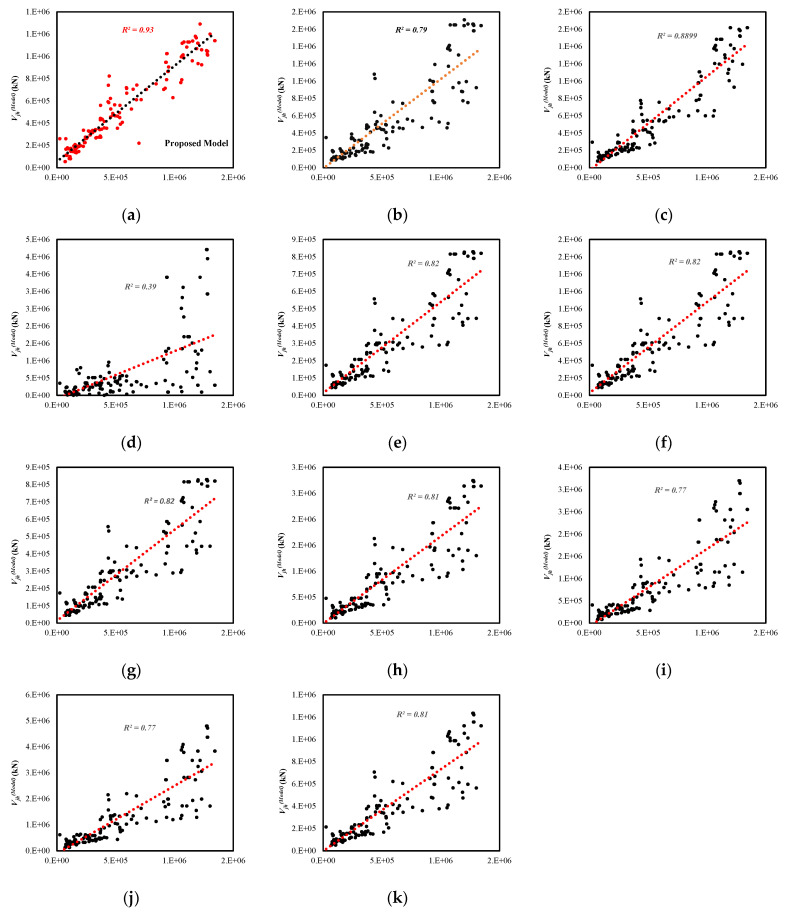
Comparison of the reinforced RC exterior joint: (**a**) Proposed Equation, (**b**) Vollum et al. [24] (**c**) Bakir et al. [4] (**d**) Montenious et al. [27] (**e**) Kim et al. [28], (**f**) ACI 352R-02 [22] (**g**) ASCE 41-17 [23] (**h**) Eurocode [18] (**i**) New Zealand code [17] (**j**) Chinese code [19] (**k**) Japanese code [20].

Table 2 compares various reinforced exterior joint models. The table shows that the proposed model’s coefficient of determination ($R^{2}$) is higher than all the other models. Correspondingly, based on the performance factor (PF) and the average absolute error (AAE), the predictive ability of the developed model is better than the other listed models. The proposed model is also shown in Figure 10 to be statistically well-founded and well-organized.

Overall, it can be concluded from Table 3 and Table 4 that the incorporation of all the key influencing parameters in the proposed model has enabled this model to accurately predict the joint shear strength developed during cyclic loading.

**Figure 11:**
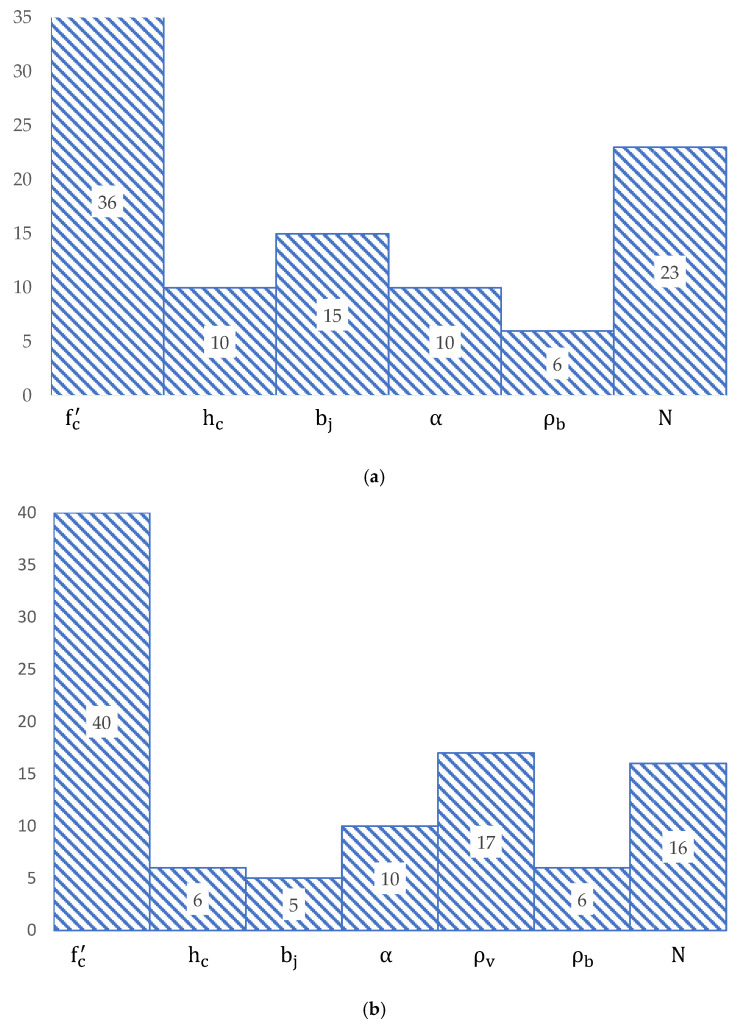
Relative contributions of key influencing variables; (**a**) unreinforced joint, (**b**) reinforced joint.

Finally, a sensitivity analysis was carried out to assess the relative contribution of the key parameters impacting the shear strength of RC joints, Figure 11. The sensitivity analysis was conducted by following the approach by Gandomi et al. [55]. According to this approach, the sensitivity ($S_{i}$) of the key variables is obtained by

(14) $$N_{i}=f_{max}\left(x_{i}\right)-f_{min}\left(x_{i}\right)$$

(15) $$S_{i}=\frac{N_{i}}{\sum_{j=1}^{n}N_{j}}\times 100$$

The $f_{max}\;\left(x_{i}\right)$ and $f_{min}$(*x_i_*) denote the respective maximum and minimum of the predicted output over the $i_{th}$ output.

From Figure 11, it is clear that both models derived for reinforced interior and exterior RC joints are very sensitive to the concrete compressive strength, as the shear strength of RC joint increases with the increase in the concrete compressive strength. After the compressive strength, the applied axial load on the column is prioritized in terms of effectiveness in both models. Notably, the increase in the applied axial load confines the joint diagonal strut, which overall enhances the shear capacity of RC reinforced and unreinforced exterior joints. The sensitivities of the other input parameters are shown in Figure 11.

## 8. Conclusions

A gene expression algorithm was used in this study to construct shear strength prediction models for the unreinforced and reinforced concrete exterior joints. These models were developed by using a large database of cyclically-loaded R.C. beam–column joints, which took into account parameters that influenced their shearing capacities, including the compressive strength of cement, the longitudinal tensile reinforcement ratio, the cross-sectional geometry of structural elements, as well as the joint aspect ratio. The new model’s prediction of the joint shear capacity was more precise than the existing models. More specifically, the average absolute error (AAE) of the current model was 38% for the unreinforced exterior joint and 25% for the reinforced joint. Similarly, the coefficient of determination ($R^{2}$) of the current model was 0.94 for the unreinforced exterior joint and 0.93 for the reinforced joints. The exterior joint performance factors were 1.10 and 1.20 for the unreinforced and reinforced joints, respectively. Additionally, the current model outperformed the shear capacity predictions as compared to various influential design codes.

Thus, the current model accurately predicts the shear capacity of the exterior joints subjected to reverse cyclic loading. Because of this, its use in the design of exterior joints can be recommended with greater confidence.

## Figures and Tables

**Table 1:** Range of input parameters.

Input Parameter	Range
$\mathrm{Compressive}\;\mathrm{strength}\;\mathrm{of}\;\mathrm{concrete}\;f_{c}^{\prime }\;$(MPa)	8.10−107.90
$\mathrm{Area}\;\mathrm{of}\;\mathrm{joint}\;\mathrm{shear}\;\mathrm{reinforcement}\;\rho_{t}\;$(mm^2^)	150.00−550.00
$\mathrm{Depth}\;\mathrm{of}\;\mathrm{column}\;h_{c}\;$(mm)	0.00−4054.00
$\mathrm{Aspect}\;\mathrm{ratio}\;\mathrm{of}\;\mathrm{the}\;\mathrm{joint}\;\left(\alpha \right)$	1.00−1.49
$\mathrm{Reinforcement}\;\mathrm{ratio}\;\mathrm{of}\;\mathrm{beam}\;(\rho_{b})$	0.003−0.107
Axial load in column (*N*) (kN)	0.00−2250.00

**Table 2:** Model construction parameters.

Chromosomes	100
Head sizes	10
Linking function	Addition
Function set	$+,-,\;/,\times ,\;sqrt,\;x^{2}$
Gene	3
Rate of mutation	0.0014
Rate of inversion	0.1
Constants per gene	10
Lower/Upper bound of constants	–20/20
One-point recombination rate	0.0027
Two-point recombination rate	0.0027
Gene recombination rate	0.0027
Gene transposition rate	0.0027

**Table 3:** Statistical calculations of the unreinforced RC exterior joint.

*Author*	$PF=\frac{v_{jh}^{\;\;Exp}}{v_{jh}^{\;\;Est}}$	*AAE* (%)	$R^{2}$
	*Mean*		
Vollum et al. [24]	1.80	89.00	0.79
Bakir et al. [4]	1.20	45.10	0.88
Montenious et al. [27]	0.64	161.50	0.50
Kim et al. [28]	0.27	323.00	0.72
ACI 352R-02 [22]	0.61	126.90	0.73
ASCE 41-17 [23]	1.23	45.80	0.72
EN 1998-1-2004 [18]	0.49	174.40	0.75
GB 50010-2011 [19]	0.39	241.70	0.72
NZS 3101-2006 [17	0.58	130.20	0.72
AIJ (1999) [20]	1.20	50.90	0.73
**Proposed**	**1.20**	**38.00**	**0.93**

**Table 4:** Statistical calculations of the reinforced RC exterior joint.

*Author*	$PF=\frac{v_{jh}^{\;\;Exp}}{v_{jh}^{\;\;Est}}$	*AAE* (%)	$R^{2}$
	*Mean*		
Vollum et al. [24]	1.20	40.00	0.79
Bakir et al. [4]	1.20	30.10	0.89
Montenious et al. [27]	3.49	76.00	0.39
Kim et al. [28]	1.90	49.00	0.81
ACI 352R-02 [22]	0.95	43.00	0.82
ASCE 41-17 [23]	1.90	49.00	0.82
EN 1998-1-2004 [18]	0.66	84.00	0.81
GB 50010-2011 [19]	0.49	151.09	0.77
NZS 3101-2006 [17	0.74	74.00	0.77
AIJ (1999) [20]	1.49	37.00	0.81
**Proposed**	**1.10**	**25.0**	**0.94**

## Data Availability

Not applicable.

## Appendix A

**Table A1:** Previously Proposed Model for RC Exterior Joint.

Vollum and Neman [24]	$V_{n}=0.642\zeta \left[1+0.552\left(2-\frac{h_{b}}{h_{c}}\right)\right]bj\;h_{c}\sqrt{fc^{\prime }}$
Bakir and Boduroglu [4]	$V_{n}=\frac{0.71\zeta {\left(100\rho_{b}\right)}^{0.4289}}{{\left(\frac{h_{b}}{h_{c}}\right)}^{0.61}}b_{j}h_{c}\sqrt{{f_{c}}^{\prime }}$
Hegger et al. [25]	$V_{n}=2\zeta \left(1.2-0.3\frac{h_{b}}{h_{c}}\right)\left(1+\frac{\rho_{c}-0.5}{7.5}\right)b_{j}h_{c}\sqrt[{3}]{f_{c}^{\prime }}$
Sarsam and Phipps [26]	$V_{n}=5.08{\left(f_{cu}\rho_{c}\right)}^{0.33}{\left(\frac{d_{c}}{d_{b}}\right)}^{1.33}b_{c}d_{c}\sqrt{1+\frac{0.29P_{c}}{A_{g}}}+0.87A_{js}f_{yv}$
Parra-Montesinos and Wight [27]	$V_{n}=\alpha_{1}\alpha_{2}f_{c}^{\prime }b_{j}h_{c}$ $\alpha_{1}=0.34-0.00018k_{s}$ $\alpha_{2}=0.00018f_{c}^{\prime }-0.03f_{c}^{\prime }+1.7$
Kim et al. [28]	$V_{n}=v_{n}b_{j}h_{c}$ $v_{n}=\alpha_{1}\beta_{1}\eta_{1}\lambda_{1}{\left(JI\right)}^{0.15}{\left(BI\right)}^{0.3}{\left(f_{c}^{\prime }\right)}^{0.75}$ $\eta_{1}={\left(1-\frac{e_{b}}{b_{c}}\right)}^{0.67}$ $JI=\frac{\rho_{y}f_{yj}}{f_{c}^{\prime }}\geq 0.0139$
Yasmin [46]	$V_{n}=\left[\left(\left(\sqrt{f_{c}^{\prime }}+7.42\right)-f_{c}^{\prime }{}^{\frac{1}{4}}\right)\times \left(-7.42\times \left(b_{j}+12.75\right)\right)\right]101225\times [\rho_{sb}\times {\left(\left(-126.72A_{sj}-b_{j}^{2}\right)-\left(\;h_{c}+P\right)\right]}^{\frac{1}{3}}$ $V_{jh}=\left[\left(-15.347\left(6.18-\sqrt{f_{c}^{\prime }}\right)\right)\times \left(\left(-2.49+\sqrt{f_{c}^{\prime }}\right)+\left(h_{c}+b_{j}\right)\right)\right]\times \left[\frac{1}{\sqrt{f_{c}^{\prime }}-4.98}-\left(\sqrt{f_{c}^{\prime }}+b_{j}\right)-\left(91.6-\left(A_{sj}-\sqrt{f_{c}^{\prime }}\right)\right)\right]$
ACI 352R-02 (2010) [22]	$\phi V_{n}\geq V_{u}$ $V_{n}=0.083\;\gamma_{c}\;\sqrt{{f_{c}}^{\prime }}\;A_{j}$ (MPa) $V_{n}=\gamma_{c}\;\sqrt{{f_{c}}^{\prime }}\;A_{j}$ (psi)
ASCE 41-17 [23]	$\gamma_{c}=6$
New Zealand design standard (NZS 3101-2006) [18]	$V_{jh}\leq \min\left[0.2f_{c}^{\prime }b_{j}h_{c};10b_{j}h_{c}\right]$
Chinese seismic standards (GB 50011:2010) [17]	$V_{jh}\leq \frac{1}{\gamma_{RE}}\left(0.3\eta_{j}\beta_{c}f_{c}b_{j}h_{j}\right)$
Eurocode 8 (EN 1998-1:2004) [19]	$V_{jhd}=\lambda \eta f_{cd}\sqrt{\left(1-\frac{v_{d}}{\eta }\right)}b_{j}h_{jc}$ $\eta =0.6\left(1-\frac{f_{ck}}{250}\right)$
Architectural Institute of Japan (AIJ) [20]	$V_{ju}=k\phi F_{j}b_{j}D_{j}$ $F_{j}=0.8f_{c}^{\prime }{}^{0.7}$ $V_{ju}=0.272f_{c}^{\prime }{}^{0.7}b_{j}D_{j}$

## Appendix B. Simple Example

An example of the specimens A1 and T1 of Bindhu et al. [76] is detailed herein to assess the predictive ability of the developed model of an RC exterior joint. The material and structural details of the specimen are given in table in Appendix B.

**Table A2:** Experimental Validation of Proposed Model.

Authors and Year	Specimen Name	Cylinder Concrete Compressive Strength (MPa)	Joint Shear Reinforcement Area (mm^2^)	Column Depth (mm)	Joint Width (mm)	Joint Aspect Ratio	Beam Reinforcement Ratio	Experimental Joint Shear Strength (kN)	Predicted Joint Experimental Strength (kN)
BINDHU and JAYA 2008 [77]	A1	36.70	396.00	150.00	100.00	1.00	0.11	74.71	68.47
	T1	23.90	0.00	200.00	200.00	1.65	0.02	62.29	59.56

Note: The experiment shown in this table has neither been used in the GEP model generation nor the model validation.
